# Abnormal Development of Tapetum and Microspores Induced by Chemical Hybridization Agent SQ-1 in Wheat

**DOI:** 10.1371/journal.pone.0119557

**Published:** 2015-03-24

**Authors:** Shuping Wang, Gaisheng Zhang, Qilu Song, Yingxin Zhang, Zheng Li, Jialin Guo, Na Niu, Shoucai Ma, Junwei Wang

**Affiliations:** 1 College of Agronomy, Northwest A&F University, National Yangling Agricultural Biotechnology & Breeding Center, Yangling Branch of State Wheat Improvement Centre, Wheat Breeding Engineering Research Center, Ministry of Education, Key Laboratory of Crop Heterosis of Shaanxi Province, Yangling, China; 2 Institute of Genetics and Developmental Biology, Chinese Academy of Sciences, Beijing, China; United States Department of Agriculture, UNITED STATES

## Abstract

Chemical hybridization agent (CHA)-induced male sterility is an important tool in crop heterosis. To demonstrate that CHA-SQ-1-induced male sterility is associated with abnormal tapetal and microspore development, the cytology of CHA-SQ-1-treated plant anthers at various developmental stages was studied by light microscopy, scanning and transmission electron microscopy, in situ terminal deoxynucleotidyl transferasemediated dUTP nick end-labelling (TUNEL) assay and DAPI staining. The results indicated that the SQ-1-treated plants underwent premature tapetal programmed cell death (PCD), which was initiated at the early-uninucleate stage of microspore development and continued until the tapetal cells were completely degraded; the process of microspore development was then blocked. Microspores with low-viability (fluorescein diacetate staining) were aborted. The study suggests that premature tapetal PCD is the main cause of pollen abortion. Furthermore, it determines the starting period and a key factor in CHA-SQ-1-induced male sterility at the cell level, and provides cytological evidence to further study the mechanism between PCD and male sterility.

## Introduction

Wheat hybrids have had a significant improvement on grain yield through improved cultivars and have led to better adaptation to adverse environments [1,2]. A number of approaches have been proposed to avoid self-pollination for the commercial production of hybrid wheat seeds, which include genetic male sterility (GMS), cytoplasmic male sterility (CMS), photo-thermo-sensitive male sterility (PTMS) and chemical hybridizing agents (CHAs) [3–6]. Of these, CHA-induced male sterility can provide rapid, flexible and high performance seed-producing female parents for F1 hybrid production; they simultaneously avoid fluctuations of genotype and environmental factors in maintaining male-sterility and/or male-fertility restoration [7]. Specifically, CHA-induced male sterility removes the reproductive isolation necessary in other approaches, the conversion and maintenance of an androsterile line (line A) and the incorporation of factors of fertility restoration in male progenitors, thus facilitating a ‘two-line’ approach to the production of hybrid seed [8,9]. CHA-induced male sterility also provides ideal material for the study of reproductive growth, cytoplasmic inheritance and pollen development.

The establishment of a highly effective, low-pollution CHA approach is critical to the utilization of heterosis. SQ-1 is a new type of CHA for use on wheat, which has broad-spectrum properties and can lead to complete male sterility as has been proven in pharmacodynamic field experiments; furthermore, it has no side effects on agronomic traits. This substantially reduces the time required for hybrid development. SQ-1 has now been used to produce hybrid seeds on a large scale in China. However, the mechanism by which CHA-SQ-1 causes male sterility in wheat is still unknown.

Pollen abortion is a key characteristic of male sterility and can be observed at various stages of pollen development in male-sterile lines. Laser and Lersten [10] list reports of abortive pollen in 38 species from 13 families of angiosperm. In dicotyledonous plants, 27% of abortions occurred during at the early stages of meiosis, 58% at the tetrad stage and 15% at the microspore developmental stage. In monocotyledonous plants 4% occurred during early meiosis, 57% at the tetrad stage and 39% at the microspore developmental stage. Subsequently, according to pollen development abort at a particular nucleate stage [11]. Pollen abortion can be classified into four types: pollen-free, uninucleate abortive, binucleate abortive and trinucleate abortive [11].

Recently, there have been a large number of reports of male sterility associated with disturbances of tapetal development and degeneration [12–19]. The anther tapetum is the innermost of the four sporophytic layers of the anther wall; it comes into direct contact with the developing male gametophyte and contains all the nutrients for microspore development and maturation, such as callose, sporopollenin and proteins [16,20–22]. Studies have proven that selective tapetal destruction can lead to pollen abortion [23,24]. Moreover, in transgenic tobacco, premature callose wall degeneration causes male sterility [25]. Another study show that CHA-RH007 treated wheat prevents the formation of sporopollen causing pollen abortion [26], and the results have been confirmed in our previous study [27]. It is known that male sterility is associated with premature tapetal programmed cell death (PCD), which has been described in PET-CMS of sunflower [28], HL-CMS of rice [29], and *TAZ1-*silenced plants [30]. Additionally, the present results demonstrate that tapetum-specific genes control tapetal development and affect pollen development [31,32], such as PR10 [33], RTS [34], TA29 [25,35,36] and TAZ1 [30]. However, little is known about the tapetal and pollen development in CHA-SQ-1-induced male sterility.

In this study, we investigated the characteristics of CHA-SQ-1-induced male sterile in wheat plants, and compared the morphological changes between normal fertile anthers from control plants and male sterile anthers from CHA-SQ-1-treated plants at different developmental stages; comparisons were made using light microscopy, electron microscopy and the biochemical characteristics of nuclear DNA fragmentation using terminal deoxynucleotidyl transferase-mediated dUTP nick end-labelling (TUNEL) assay in situ. Here we show that CHA-SQ-1-induced male sterility is associated with premature tapetal PCD, resulting in failure to form mature pollen grains. Detailed premature PCD in the anther tapetum is also discussed.

## Material and Methods

### Ethics Statement

No specific permissions were required for the described field studies and for the location and activities. The location is not privately-owned or protected in any way. The field studies did not involve endangered or protected species.

### Chemicals

SQ-1, a new pyridazine compound, was provided by Key Laboratory of Crop Heterosis of Shaanxi Province, and its main ingredient is 4-chloroaniline. SQ-1 can induce complete male sterility in wheat when applied at a concentration about 5.0 kg ha^-1^.

### Plant material and treatment

The details of wheat treatment have been described previously [4–6,37]. The wheat cultivar ‘Xinong 1376’, induced by SQ-1 at the rate of 5.0 kg ha^-1^, was sprayed when the wheat averaged 8.5 stage on the Feekes’ scale. All plants were grown conventionally in wheat fields of the experimental station of Northwest Agriculture and Forestry University, Yangling, China (108° E, 34° 15′ N). The microspore developmental stages were checked as described previously [4–6,37].

### Phenotypic characterization

Plant materials were photographed with a Nikon E995 digital camera (Nikon, Japan) mounted on a Motic K400 dissecting microscope (Preiser Scientific, Louisville, KY, USA). Fresh microspores were stained with 1% acetocarmine and 2% iodine-potassium iodide (2% I_2_-KI). Anthers at different developmental stages were fixed in FAA and embedded in paraffin wax. Transverse sections of 6 μm were placed onto poly-L-lysine-coated slides (Sigma-Aldrich), and stained with safranin O/fast green and Ehrlich’s hematoxylin. For 4’, 6-diamidino-2-phenylindole (DAPI) staining of the nuclei, transverse sections and microspores were washed, embedded, and stained as described previously [37]. Samples were photographed using a DS-U2 high resolution camera mounted on a Nikon ECLIPSE E600 microscope along with NIS-Elements software (all from Nikon).

For transmission electron microscopy observation, anthers were fixed, embedded, and stained according to Cheng et al. [7] and examined with a JEM-1230 transmission electron microscope (JEOL). For scanning electron microscopy, anthers and microspores were collected and processed essentially as described by Zhang et al. [38], and observed with a JSM-6360LV scanning electron microscope (JEOL).

### TUNEL (Tdt-mediated dUTP nick-end labelling) assay

TUNEL assay was performed using a Dead End Fluorometric TUNEL Kit (Promega, Madison, WI, USA), following the manufacturer’s instructions. Transverse sections were washed in PBS for 5 min and fixed in 4% (w/v) paraformaldehyde in PBS for 15 min, and then incubated in 20 μg mL^-1^ Proteinase K (Promega) in PBS for 10 min. In situ nick-end labelling of nuclear DNA fragmentation was performed in a humid chamber for 60 min at 37°C in the dark. The positive controls were incubated in 1 U μL^-1^ DNase I (Promega) for 10 min at 37°C before labelling as above. The negative controls were labelled in parallel, except for the absence of the enzyme terminal deoxynucleotidyl transferase (TdT). All reactions were counterstained with 1μg mL^-1^ of propidium iodide (PI; Promega) in PBS for 15 min to label nuclei. Samples were analysed using a fluorescence confocal scanner microscope (A1R; Nikon, Tokyo, Japan). The green fluorescence of fluorescein (TUNEL signal) and red fluorescence of PI were analysed at 488 nm (excitation) and 520 nm (detection), and at 488 nm (excitation) and 620 nm (detection), respectively.

### FDA (fluorescein diacetate) staining

Fresh anthers were embedded in optimal cutting temperature medium (Sakura Finetek, Torrance, CA, USA), frozen and sectioned. Sections of 10 μm were placed onto DNase and RNase free slides. Meanwhile, fresh microspores were squeezed out into an Eppendorf tube. Sections and microspores were washed with PBS and then incubated in 10 μg mL^-1^ fluorescein diacetate (FDA; Sigma-Aldrich) in the dark to maximize formation of fluorescein. The fluorescent FDA signals were detected with a fluorescence microscope (Olympus BX 51, Olympus, Japan) by monitoring emission at 520 nm (green) with an excitation wavelength of 485 nm. Only cells that exhibited bright green fluorescence from their cytosol were considered to be viable.

## Results

### Morphological features of CHA-SQ-1 induced male sterile wheat

**Fig 1:**
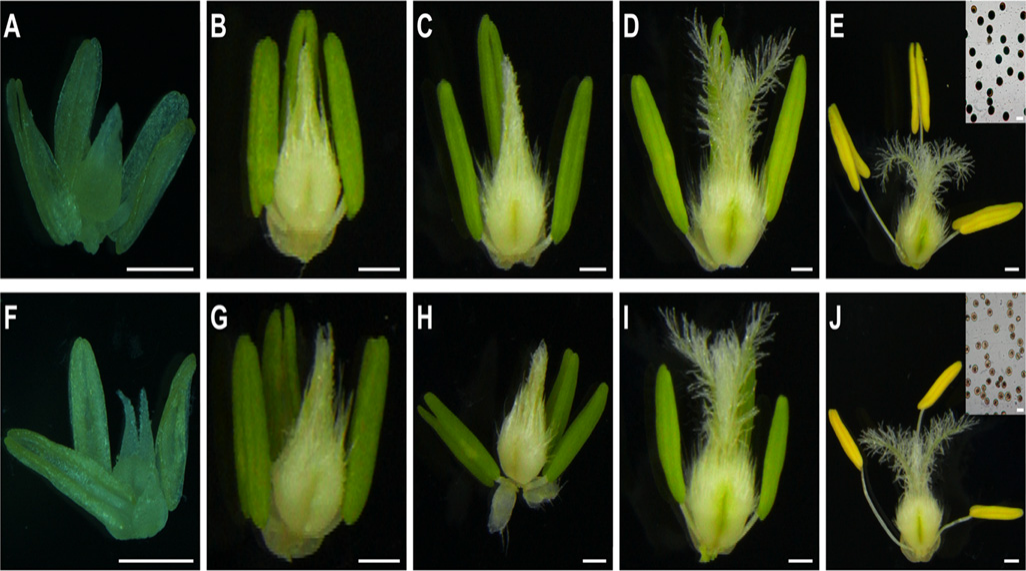
Comparison of stamens and pistils of untreated (A to E) and chemical hybridization agent (CHA)-SQ-1-treated wheat plants (F to J). (A and F) the tetrad stage. (B and G) the early-uninucleate stage. (C and H) the later-uninucleate stage. (D and I) the binucleate stage. (E and J) the trinucleate stage. (E and J, top right) the 2% I_2_-KI (top right) staining pollen grains. Scale bars are 0.5 mm in A to J and are 50 μm in the top right of E and J.

**Fig 2:**
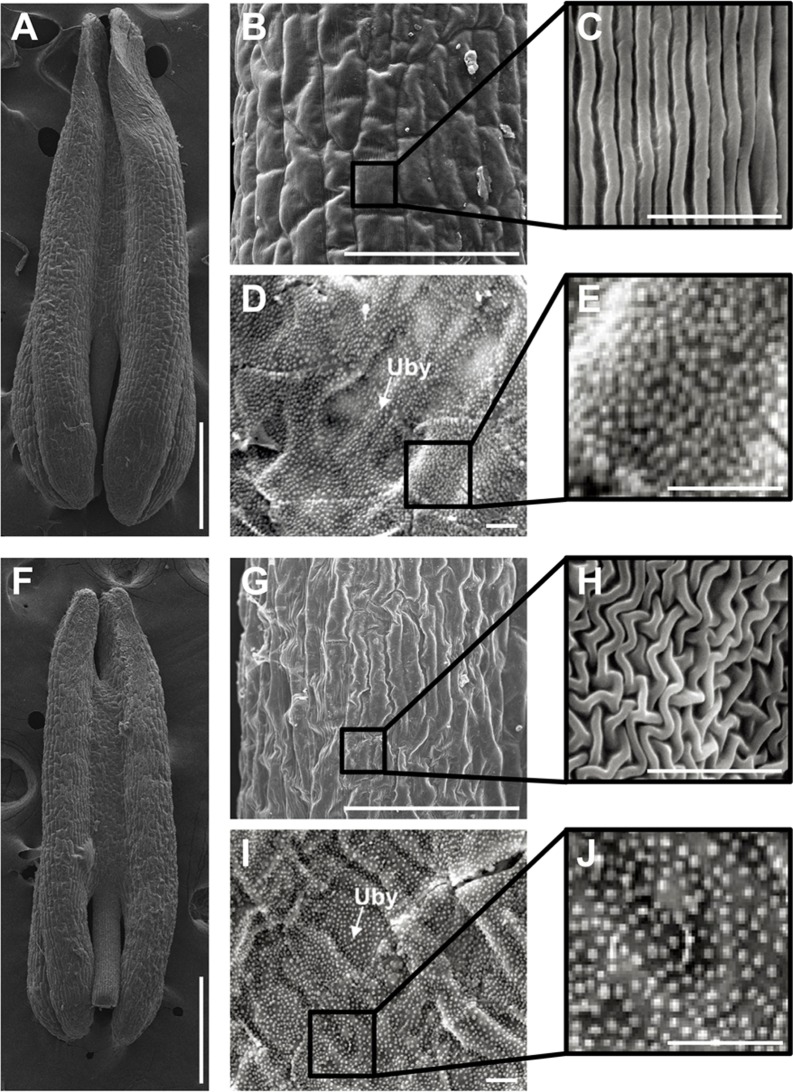
Scanning electron micrograph observations of untreated (A to E) and chemical hybridization agent (CHA)-SQ-1-treated wheat plant (F to J) anthers at the trinucleate stage. (B, C, G and H) the outer epidermal cells. (D, E, I and J) the inner epidermal cells. Uby indicate the Ubisch body. Scale bars are 1 mm in A and F and are 100 μm in B and G and are 10 μm in C to E and H to J.

Based on morphological landmarks or cellular events visible under the light microscope and previous classification of anther development [4–6,37], we divided wheat anther development into five stages. The CHA-SQ-1-treated plant anthers appeared normal at the tetrad stage (Fig. 1A,F). However, from the early-uninucleate stage to the trinucleate stage, the anthers of treated plants were smaller than those of untreated plants (Figs. 1B–E,G–J and 2A,F). More importantly, at the trinucleate stage, unlike untreated mature pollen, the treated pollen could not be deeply stained with iodine-potassium iodide (2% I_2_-KI), and the plants were 100% pollen sterile (Fig. 1E,J, top right). Moreover, treated plant pistils showed normal development (Fig. 1) and were able to produce normal seeds when backcrossed with fertile pollen.

To gain a more detailed understanding of the abnormalities of the CHA-SQ-1-treated plant anthers at the trinucleate stage, we used scanning electron microscopy (SEM) to study the outer and inner epidermal surfaces of anther pattern (Fig. 2). The anther outer epidermal cells appeared to be smaller and more shrunken than in untreated plant cells (Fig. 2B,G) and were irregularly shaped (Fig. 2C,H). Meanwhile, the inner epidermal cells accumulated less Ubisch bodies (Fig. 2D,E,I,J).

The above results indicated that SQ-1 had a major impact on the development of the anther and that this could induce complete male sterility.

### Effects of CHA-SQ-1 on the development of tapetum and microspores

**Fig 3:**
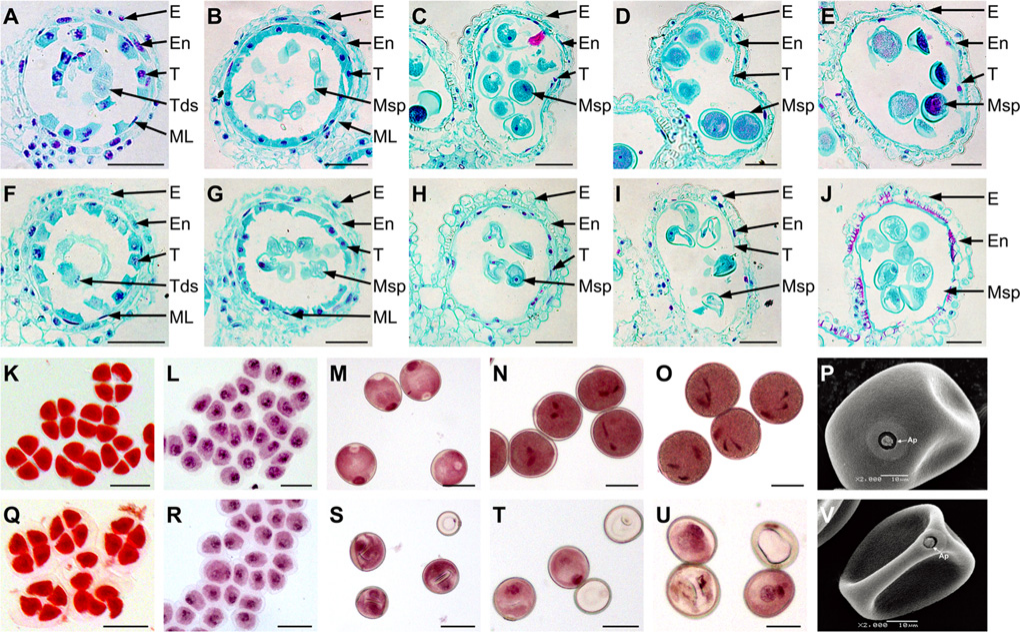
Development of anthers and microspores in untreated (A to E and K to P) and chemical hybridization agent (CHA)-SQ-1-treated wheat plants (F to J and Q to V). (A to J) safranin O/fast green-stained transverse sections. (K to O and Q to U) 1% acetocarmine-stained microspores. (P and V) a scanning electron micrograph was used to analyse the mature pollen grains. (A, F, K and Q) the tetrad stage. (B, G, L and R) the early-uninucleate stage. (C, H, M and S) the later-uninucleate stage. (D, I, N and T) the binucleate stage. (E, J, O and U) the trinucleate stage. E, En, ML, T, Tds, Msp and Ap indicate the epidermis, the endothecium, the middle layer, the tapetum, the tetrads, the microspore and the germination aperture, respectively. Scale bars are 50 μm in A to O and Q to U.

To further investigate cytological structural defects, we performed a detailed examination of untreated and CHA-SQ-1-treated plant anther development using light microscopy (Figs. 3 and S1) and transmission electron microscopy (S2 Fig.).

At the tetrad stage, the tapetal cells of treated plants were significantly larger than any other stage and contained within an agglomerate which could not be disaggregated; the cells possessed dense cytoplasm and could be deeply stained with safranin O/fast green (Fig. 3A–J) and Ehrlich’s hematoxylin (S1 Fig.). In addition, the middle layer assumed a band-like shape. There was no obvious difference in anther cellular morphology between untreated and CHA-SQ-1-treated plants. Normal epidermis, endothecium, middle layer, and tapetum were found in both untreated and CHA-SQ-1-treated plants (Fig. 3A,F; S2A,F Fig.). Tetrads of haploid microspores proceeded normally and could undergo meiosis to form uninucleate microspores (Fig. 3K,Q). During the early-uninucleate stage, the untreated plant tapetal cells remained relatively thick (S2B Fig.) and their cytoplasm could be deeply stained (Figs. 3B; S1B Fig.). By contrast, the CHA-SQ-1-treated plant tapetal cells were not as deeply stained as that of the untreated plant tapetum, which became narrower (S2G Fig.) and began to degenerate (Fig. 3G). Meanwhile, the middle layers became very thin and were still clearly visible in both untreated and CHA-SQ-1-treated plants (Fig. 3B,G; S2B,G Fig.). Microspores were released from tetrads and their development seemed normal (Fig. 3L,R). Up to the later-uninucleate stage, the middle layers in both treated and untreated plants were hardly visible (Figs. 3C,H, and S2C,H). The epidermis and endothecium of untreated plants became thin, and the tapetum began to degenerate forming a hill-like shape; however, most of the tapetal cells still had a nuclear contour and relatively abundant cytoplasm (Figs. 3C and S2C). These processes are crucial for supplying nutrition to the developing microspores. At this stage, all microspores appeared to have germination apertures and were round, they also contained an enormous vacuole and displayed an increased microspore volume; the nucleus was displaced to the opposite side from the germination aperture (Fig. 3M). In contrast, CHA-SQ-1-treated plants displayed delayed degradation of the epidermis and endothecium (Fig. 3H), and produced severely abnormal microspores with a typical falcate (sickle-like) shape or that had become highly vacuolated (Fig. 3S). At the binucleate stage, the untreated plant tapetal cells remained relatively thick with an integral narrow-band-like shape (Fig. 3D and S2D). Microspores continued to increase in volume and began to accumulate nutrients; they had a dense cytoplasm and two distinct nuclei which were visible by staining with 1% acetocarmine (Fig. 3N). However, the tapetal cells from treated plants disintegrated into debris (Figs. 3I and S2I), and most of the microspores failed to develop and were degraded (Fig. 3T). At the trinucleate stage, the untreated plant tapetal cell outlines remained clear (S2E Fig.) but the anther wall layers were thinner (Fig. 3E); this contributed to mature pollen grains being released by anther dehiscence to pollinate female gametophytes. The mature pollen grains were clearly distinguished by two sperm nuclei and nuclear nutrients including a full complement of starch, lipids, and other storage materials (Fig. 3O,P) which are important for microspore viability and function. However, though the treated plant tapetum were fully degraded and invisible (S2J Fig.), the epidermis and endothecium were thicker than normal at this stage (Fig. 3J), which produced severely abnormal microspores (Fig. 3U). Moreover, SEM analysis further confirmed that the CHA-SQ-1-treated plants displayed a completely misshapen and shrunken extine pattern (Fig. 3V).

These observations suggest that SQ-1-treated plants developed defects in tapetal degeneration, further affecting microspore development.

### Detection of TUNEL and DNA fragmentation

**Fig 4:**
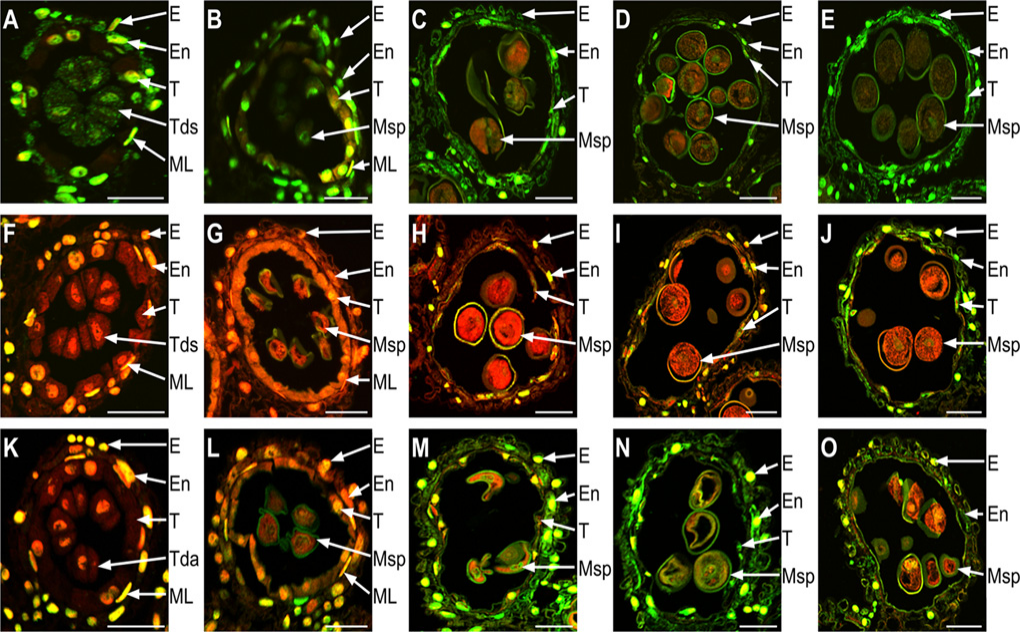
DNA fragmentation (indicating programmed cell death; PCD) in untreated and chemical hybridization agent (CHA)-SQ-1-treated wheat plants during anther development. Anther sections in the DNase treatment (Positive control, A to E), untreated (F to J) and CHA-SQ-1-treated plants (K to O) were compared for nuclear DNA fragmentation using the TUNEL assay at the tetrad stage (A, F and K), early-uninucleate stage (B, G and L), later-uninucleate stage (C, H and M), binucleate stage (D, I and N) and trinucleate stage (E, J and O), respectively. Propidium iodide stained nuclei fluoresce red, while green fluorescence is TUNEL-positive nuclei staining. E, En, ML, T, Tds and Msp indicate the epidermis, the endothecium, the middle layer, the tapetum, the tetrads and the microspore, respectively. Scale bars are 50 μm.

The phenotypic analysis described above suggests that SQ-1 treatment of plants affected the degradation of tapetal cells and microspore development. In plants, tapetal degeneration is considered to be the result of PCD, which is characterized by the cleavage of nuclear DNA. To further investigate the abnormal degeneration observed in the anthers of CHA-SQ-1-treated plants, cleavage of nuclear DNA was studied using TUNEL assay (Fig. 4) and DAPI staining (S3 Fig.) on anther transverse sections and a range of developmental stages were analysed. For the experimental positive control, the assay was performed with sections prepared from fertile anthers, after treatment of these sections with DNase to induce DNA fragmentation (Fig. 4A–E). At the tetrad stage, both untreated and CHA-SQ-1-treated plant tapetal cells showed TUNEL-negative nuclei (Fig. 4F,K), indicating a lack of DNA fragmentation at this stage. At the early-uninucleate stage, a TUNEL fluorescence signal was still not detected in untreated plants (Fig. 4G). However, a weak TUNEL fluorescence signal was detected in CHA-SQ-1-treated plant tapetal cells, suggesting that PCD had occurred in the tapetum (Fig. 4L), but this was not detectable in DNA ladders (S4 Fig.). Subsequently, from the later-uninucleate stage to the binucleate stage, few fragmented DNA signals were observed in untreated plant tapetal cells (Fig. 4H,I). By contrast, treated cells showed stronger TUNEL-positive signals (Fig. 4M,N), which indicated a notable accumulation of DNA fragmentation and this was further confirmed by DNA ladders (S4 Fig.). Up to the trinucleate stage, untreated plant tapetal cells were still observed and also showed stronger TUNEL fluorescence signals (Fig. 4J); this reflected the conditions necessary for starch accumulation in mature pollen grains and anther dehiscence (Fig. 1E, top right). However, CHA-SQ-1-treated plant tapetum degraded completely and disappeared (Fig. 4O). Although the TUNEL signals were present in other layers of the treated plant anthers (epidermis, endothecium and middle layer), from the early-uninucleate stage to trinucleate stage, we also observed that the anther walls were thicker than normal and eventually formed enormous non-nuclear cells which blocked nutrient biosynthesis and importation and thus further accelerated pollen abortion.

**Fig 5:**
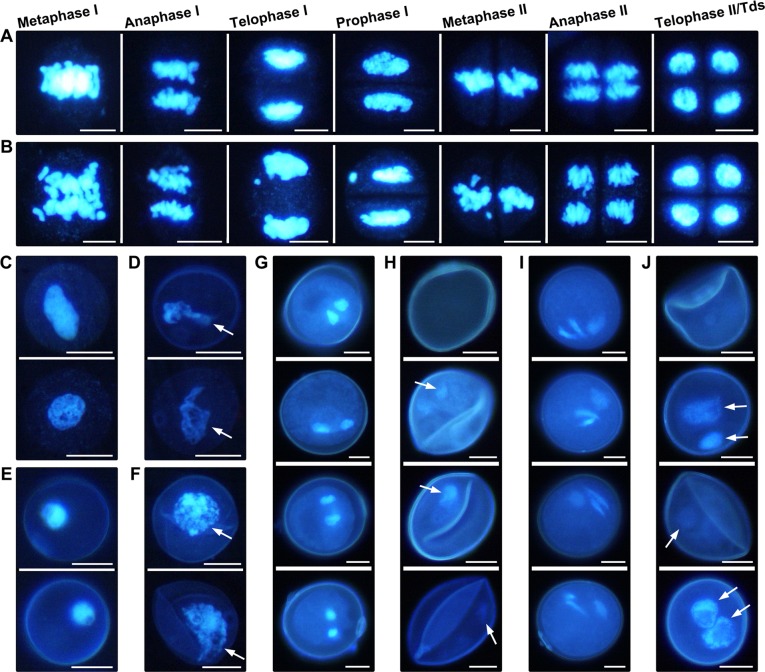
DAPI-stained showing microspore development of untreated and chemical hybridization agent (CHA)-SQ-1-treated wheat plants. Five stages of microspore development in untreated plants (A, C, E, G and I) and the corresponding stages of the CHA-SQ-1-treated plants (B, D, F, H and J) were compared. (A and B) the tetrad stage. (C and D) the early-uninucleate stage. (E and F) the later-uninucleate stage. (G and H) the binucleate stage. (I and J) the trinucleate stage. White arrows indicate abnormal nuclei. Scale bars are 10 μm.

The tapetum is considered to regulate the development of microspores by supplying nutrients to them [39,40]. To further test whether premature tapetal degradation in CHA-SQ-1-treated plants affected microspore development and what happened at different stages, we used in situ observation of microspore chromosome behaviour at different developmental stages through DNA microfluorometry with DAPI staining (Fig. 5).

On the entry into the meiotic cycle, the microsporocyte nuclei exhibited a rather distinct appearance, with their chromosomes becoming highly contracted and exhibiting brighter DAPI staining; the treated and untreated plant microsporocytes appeared to have normal development and undergo meiosis to generate tetrads of haploid microspores (Fig. 5A,B).

In the subsequent stage, it was obvious that late microspore developmental stages were abnormal in CHA-SQ-1-treated plants. From the early-uninucleate stage to the trinucleate stage, the untreated plant microspores proceeded through two rounds of mitosis to develop into mature tricellular pollen grains, and their chromosomes retained a highly contracted phenotype that exhibited brighter DAPI staining (Fig. 5C,E,G,I). By contrast, mitosis seemed to be delayed in treated plant microspores; they were irregularly shaped, and their chromosomes were loosely scattered or even absent (Fig. 5D,F,H,J).

These observations demonstrate that tapetal PCD in CHA-SQ-1-treated plants commences at the early-uninucleate stage and degrades completely at the trinucleate stage. Concomitantly, premature tapetal degradation blocks mitotic nuclear division in microspores from the early-uninucleate stage.

### The FDA viability assay for anthers

**Fig 6:**
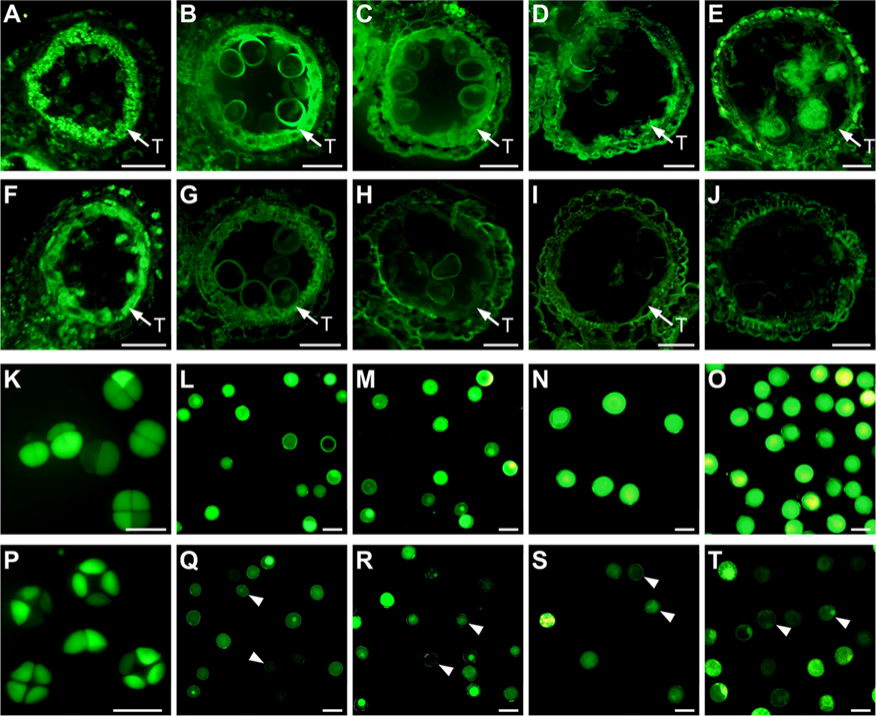
FDA-stained transverse sections and microspores of untreated and chemical hybridization agent (CHA)-SQ-1-treated wheat plants. Transverse sections (A to J) and microspores (K to T) treated with fluorescein diacetate (FDA) for the detection of cell viability and membrane integrity of untreated and CHA-SQ-1-treated plants at the tetrad stage (A, F, K and P), early-uninucleate stage (B, G, L and Q), later-uninucleate stage (C, H, M and R), binucleate stage (D, I, N and S) and trinucleate stage (E, J, O and T), respectively, as viewed under a fluorescence microscope (excitation wavelength 450–490 nm). FDA signals appear green in colour, and microspores with relatively weak or no signal were considered to be of low-viability or dead (see white *arrowhead*). The microspore survival rates are shown in S5 Fig. T indicates the tapetum. Scale bars are 50 μm.

Due to abnormal development of the tapetum and microspores in CHA-SQ-1-treated plants, the protoplasts of cells can be damaged, thereby affecting cell viability. To further elucidate microspore viability during the process of pollen abortion, we stained microspores using fluorescein diacetate (FDA; Fig. 6). Cells with intact plasma membranes and respiration showed green fluorescence; those that did not show FDA-fluorescence were considered dead. At the tetrad stage, both untreated and treated plant tapetum and microspores showed strong FDA signals (Fig. 6A,F,K,P), with high vitality (98% microspore survival in both untreated and treated plants; S5 Fig.). In the subsequent stage, the untreated tapetum and microspores retained relatively high vitality (microspore survival rate: 94.5% at the early-uninucleate stage, 93.4% at the later-uninucleate stage, 97.4% at the binucleate stage and 98.2% at the trinucleate stage; Figs. 6B–E,L–O and S5). However, the FDA signal was weaker in CHA-SQ-1-treated plants starting at the early-uninucleate stage (91.6% microspore survival) and reaching a minimum level at the trinucleate stage (29.5% microspore survival; Figs. 6G–J,Q–T and S5).

These results imply that abnormal tapetal and microspore development disrupted cell membranes and inhibited respiratory activity which resulted in visible pollen of nil or low-viability.

## Discussion

### CHA-SQ-1-treated plants abort in the early-uninucleate stage

In this study, our light microscopy observations combined with electron microscopy analysis of cytological structures indicated that various tapetal defects occurred in CHA-SQ-1-treated plants throughout the entire anther maturation process, when compared with that in control plants; furthermore, they showed premature cell degradation of the tapetum before pollen maturation. The cytological study demonstrated that the abnormal microspores first appeared at the later-uninucleate stage. During the development of the anther, tapetal cell degradation and anther dehiscence are recognized features of PCD in male sterility [16,18,41–45]; this begins with nuclear DNA fragmentation to form nucleosomes (≥180 bp), and subsequently abnormal cytological structures become visible. As the morphological characteristics of pollen abortion lag far behind its nuclear DNA fragmentation, the use of cytological observation alone delays the determination of the initial period of male sterility. Subsequently, we used TUNEL and DAPI assays to investigate the sporophytic nucleate stage to determine the accurate period of pollen abortion and discovered that the microspore development of CHA-SQ-1-treated plants was aborted at the early-uninucleate stage.

### The development pattern of the tapetum in CHA-SQ-1-treated plants

The tapetum is a specialized cell layer between the sporogenous tissue and the anther wall, which secretes all the tapetal-derived pollen coat components necessary for pollen maturation. It is clear that the degeneration of the tapetum is a very tightly controlled process, which must occur in a synchronized manner; failing to do so frequently results in sterility [46]. In light of the cytological study, although the treated plant tapetum initially appeared normal, they subsequently became abnormal, mainly through their irregular appearance, leakage of cellular contents and rapid degeneration, and eventually they disappeared at the trinucleate stage. Tapetal degeneration is not an uncontrolled event, but a process of PCD [17,28,33,38]. We used TUNEL assay and DAPI staining to further demonstrate the above abnormal processes, and showed that the SQ-1-treated plants underwent premature PCD of the tapetal cells. Additionally, anther dehiscence is also a consequence of PCD [44,45]. Similarly, in this study, we also observed that PCD was also present in controls and treatments, especially in the trinucleate stage (before anther dehiscence). But, reference to the positive control, there were distinct differences between controls and treatments, and the results were further confirmed by DNA ladder. The differences blocked nutrient biosynthesis and importation of foreign substances in SQ-1-treated plant anthers [6], and also disrupted energy metabolism [37], thereby further accelerating pollen abortion.

### The pattern of development of the microspores in CHA-SQ-1-treated plants

Production of functional pollen grain depends on timely death and degeneration of the tapetum, accelerating or delaying degeneration directly influences pollen development [11]. During normal development, the sporogenous cells divide to generate microspore mother cells, which subsequently undergo meiosis to form tetrads of haploid microspores, and then undergo two rounds of cell division to develop into mature pollen. Instead, the SQ-1-treated plants underwent premature tapetal degeneration, which limited nutrient and energy supply to the developing microspores. From the early-uninucleate stage, microspores showed abnormal development that included irregular appearance, a lack of sporopollenin, plasma membrane damage and low-viability, cytoplasm leakage and they eventually became highly vacuolated or degenerate.

Based on these reports, we believe that CHA-SQ-1-treated plants undergo early-uninucleate abortion, which causes premature tapetal PCD and consequent sporophytic male sterility. Premature PCD of the tapetum is the main cause of pollen abortion and may be associated with oxidative stress [4]. This study adds a new connotation to PCD in plants, and also opens up a new line of research involving the role of molecular biology and cell biology in mechanism of male sterility in plants.

## Supporting Information

S1 Fig Ehrlich’s hematoxylin-stained transverse sections of untreated and chemical hybridization agent (CHA)-SQ-1-treated wheat plants. (A to E) the untreated plants. (F to J) the CHA-SQ-1-treated plants. (A and F) the tetrad stage. (B and G) the early-uninucleate stage. (C and H) the later-uninucleate stage. (D and I) the binucleate stage. (E and J) the trinucleate stage. E, En, ML, T, Tds and Msp indicate the epidermis, the endothecium, the middle layer, the tapetum, the tetrads and the microspore, respectively. Scale bars are 50 μm.
(TIF)

S2 Fig Transmission Electron Micrographs of the Anthers from the untreated and chemical hybridization agent (CHA)-SQ-1-treated wheat plants. (A to E) the untreated plants. (F to J) the CHA-SQ-1-treated plants. (A and F) the tetrad stage. (B and G) the early-uninucleate stage. (C and H) the later-uninucleate stage. (D and I) the binucleate stage. (E and J) the trinucleate stage. E, En, ML, T, Tds and Msp indicate the epidermis, the endothecium, the middle layer, the tapetum, the tetrads and the microspore, respectively. Scale bars are 5 μm.
(TIF)

S3 Fig Transverse sections of wheat anthers stained with DAPI. (A to E) the untreated plants. (F to J) the CHA-SQ-1-treated plants. (A and F) the tetrad stage. (B and G) the early-uninucleate stage. (C and H) the later-uninucleate stage. (D and I) the binucleate stage. (E and J) the trinucleate stage. E, En, ML, T, Tds and Msp indicate the epidermis, the endothecium, the middle layer, the tapetum, the tetrads and the microspore, respectively. Scale bars are 50 μm.
(TIF)

S4 Fig Detection of DNA laddering in untreated and chemical hybridization agent (CHA)-SQ-1-treated wheat plant anther tissues. Total DNA isolated from wheat anthers at different developmental stages and separated by electrophoresis on a 1.8% agarose gel. Ten μg of DNA treated with RNase was loaded into each lane. Lane M indicates the DNA molecular makers. lane 1, 3, 5, 7 and 9, the untreated plants. lane 2, 4, 6, 8 and 10, the CHA-SQ-1-treated plants. lane 1 and 2, the tetrad stage. lane 3 and 4, the early-uninucleate stage. lane 5 and 6, the later-uninucleate stage. lane 7 and 8, the binucleate stage. lane 9 and 10, the trinucleate stage.
(TIF)

S5 Fig Analysis of microspore survival rate in untreated and chemical hybridization agent (CHA)-SQ-1-treated wheat plants. Microspores were stained with FDA (fluorescein diacetate) to determine cell viability and statistical analysis. Data which come from Fig. 6K to 6T and their repeated experiments images are means ± SD of three independent experiments. The significant of differences between untreated and CHA-SQ-1-treated plants were assessed by Student's t test (*P < 0.05, **P < 0.01). Td, Eun, Lun, Bn and Tn indicate the tetrad stage, the early-uninucleate, the later-uninucleate stage, the binucleate stage and the trinucleate stage, respectively.
(TIF)
