# Supplementary figures and images for: Abnormal Development of Tapetum and Microspores Induced by Chemical Hybridization Agent SQ-1 in Wheat
Source: PLoS One. 2015 Mar 24;10(3):e0119557. doi: 10.1371/journal.pone.0119557 (PMC4372346; doi:10.1371/journal.pone.0119557)

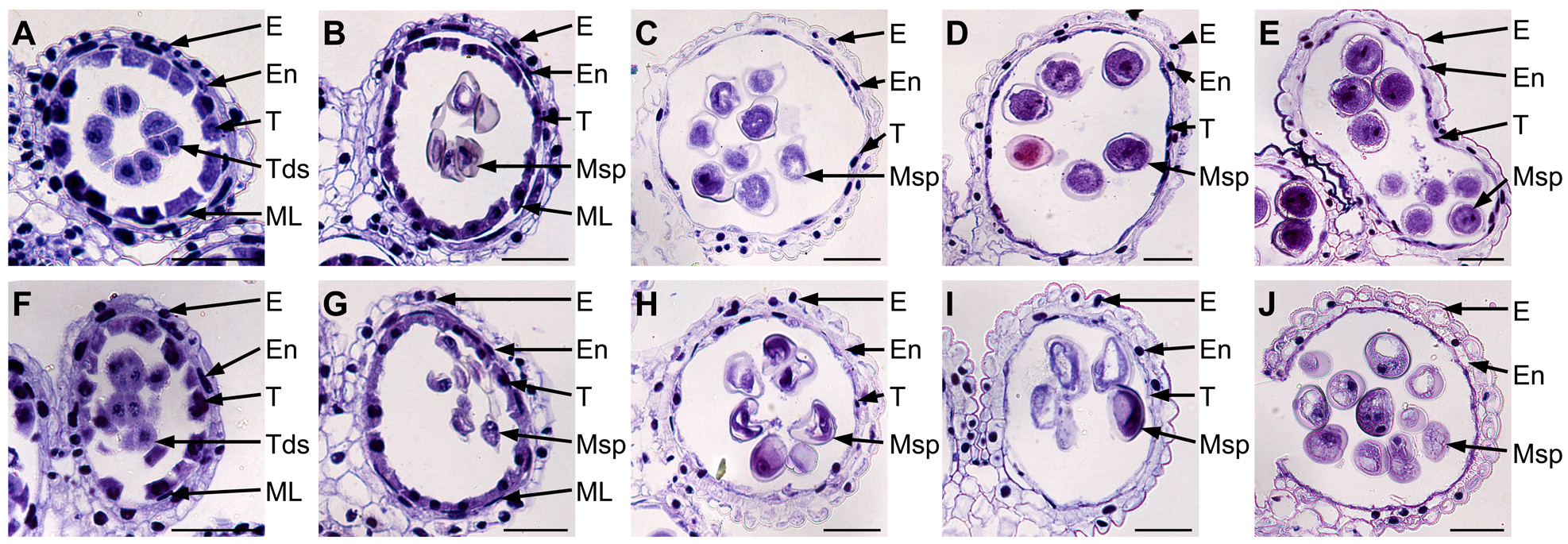

Supplement: S1 Fig — (A to E) the untreated plants. (F to J) the CHA-SQ-1-treated plants. (A and F) the tetrad stage. (B and G) the early-uninucleate stage. (C and H) the later-uninucleate stage. (D and I) the binucleate stage. (E and J) the trinucleate stage. E, En, ML, T, Tds and Msp indicate the epidermis, the endothecium, the middle layer, the tapetum, the tetrads and the microspore, respectively. Scale bars are 50 μm. (TIF) [file pone.0119557.s001.tif]

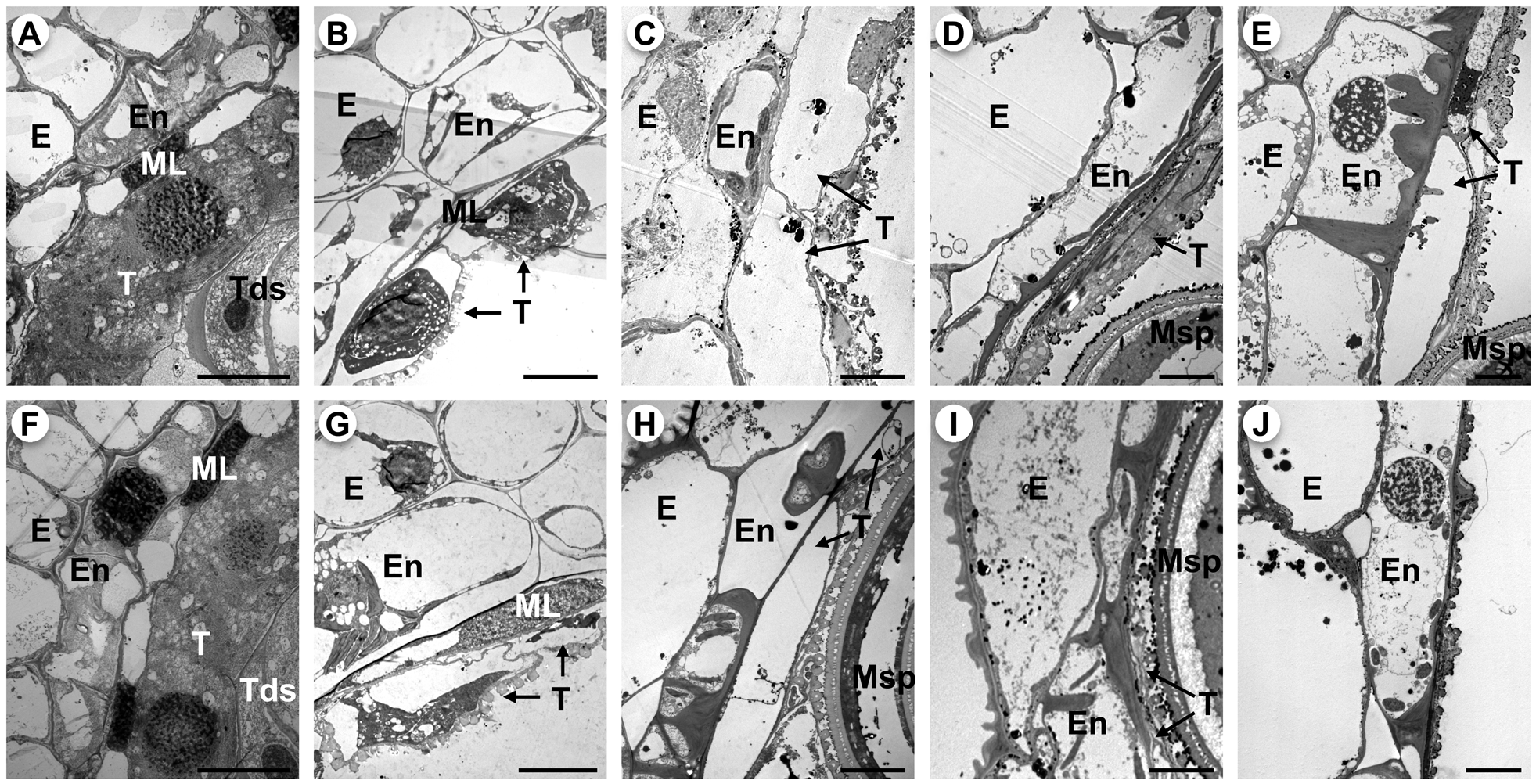

Supplement: S2 Fig — (A to E) the untreated plants. (F to J) the CHA-SQ-1-treated plants. (A and F) the tetrad stage. (B and G) the early-uninucleate stage. (C and H) the later-uninucleate stage. (D and I) the binucleate stage. (E and J) the trinucleate stage. E, En, ML, T, Tds and Msp indicate the epidermis, the endothecium, the middle layer, the tapetum, the tetrads and the microspore, respectively. Scale bars are 5 μm. (TIF) [file pone.0119557.s002.tif]

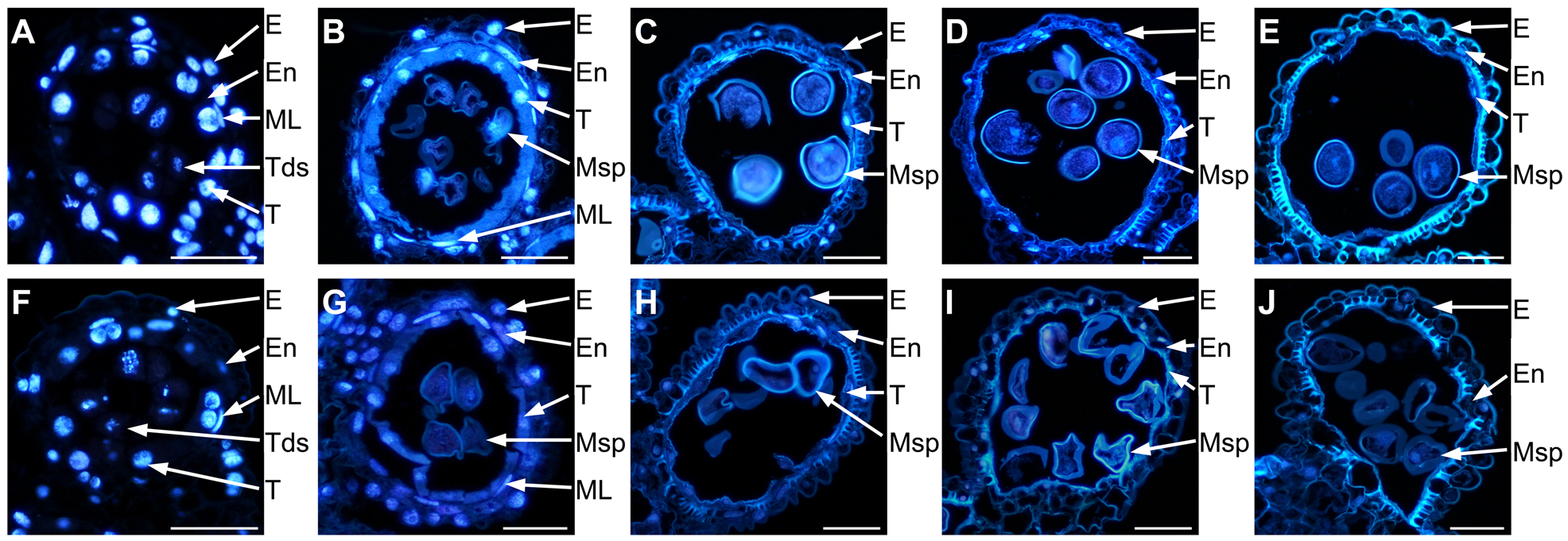

Supplement: S3 Fig — (A to E) the untreated plants. (F to J) the CHA-SQ-1-treated plants. (A and F) the tetrad stage. (B and G) the early-uninucleate stage. (C and H) the later-uninucleate stage. (D and I) the binucleate stage. (E and J) the trinucleate stage. E, En, ML, T, Tds and Msp indicate the epidermis, the endothecium, the middle layer, the tapetum, the tetrads and the microspore, respectively. Scale bars are 50 μm. (TIF) [file pone.0119557.s003.tif]

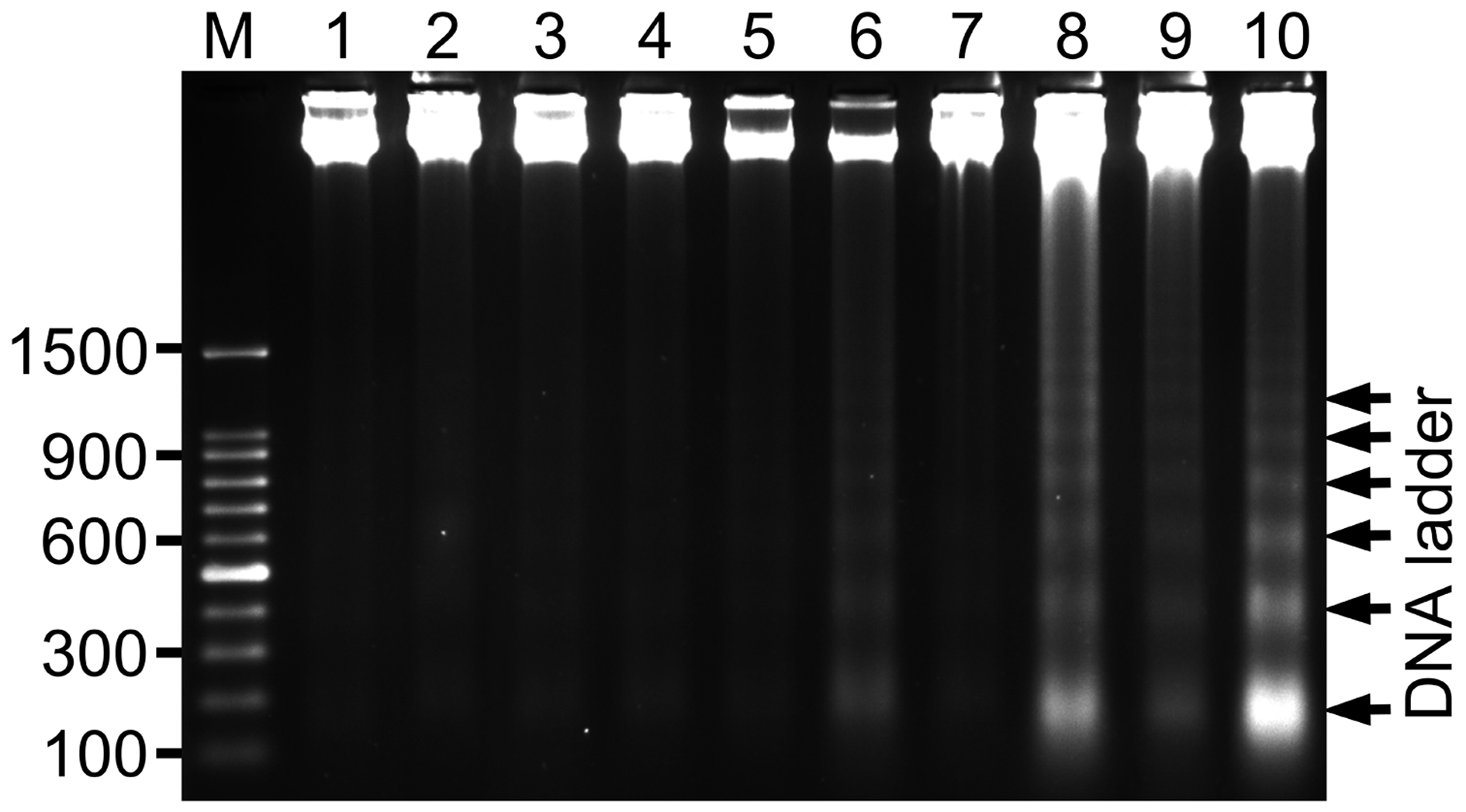

Supplement: S4 Fig — Total DNA isolated from wheat anthers at different developmental stages and separated by electrophoresis on a 1.8% agarose gel. Ten μg of DNA treated with RNase was loaded into each lane. Lane M indicates the DNA molecular makers. lane 1, 3, 5, 7 and 9, the untreated plants. lane 2, 4, 6, 8 and 10, the CHA-SQ-1-treated plants. lane 1 and 2, the tetrad stage. lane 3 and 4, the early-uninucleate stage. lane 5 and 6, the later-uninucleate stage. lane 7 and 8, the binucleate stage. lane 9 and 10, the trinucleate stage. (TIF) [file pone.0119557.s004.tif]

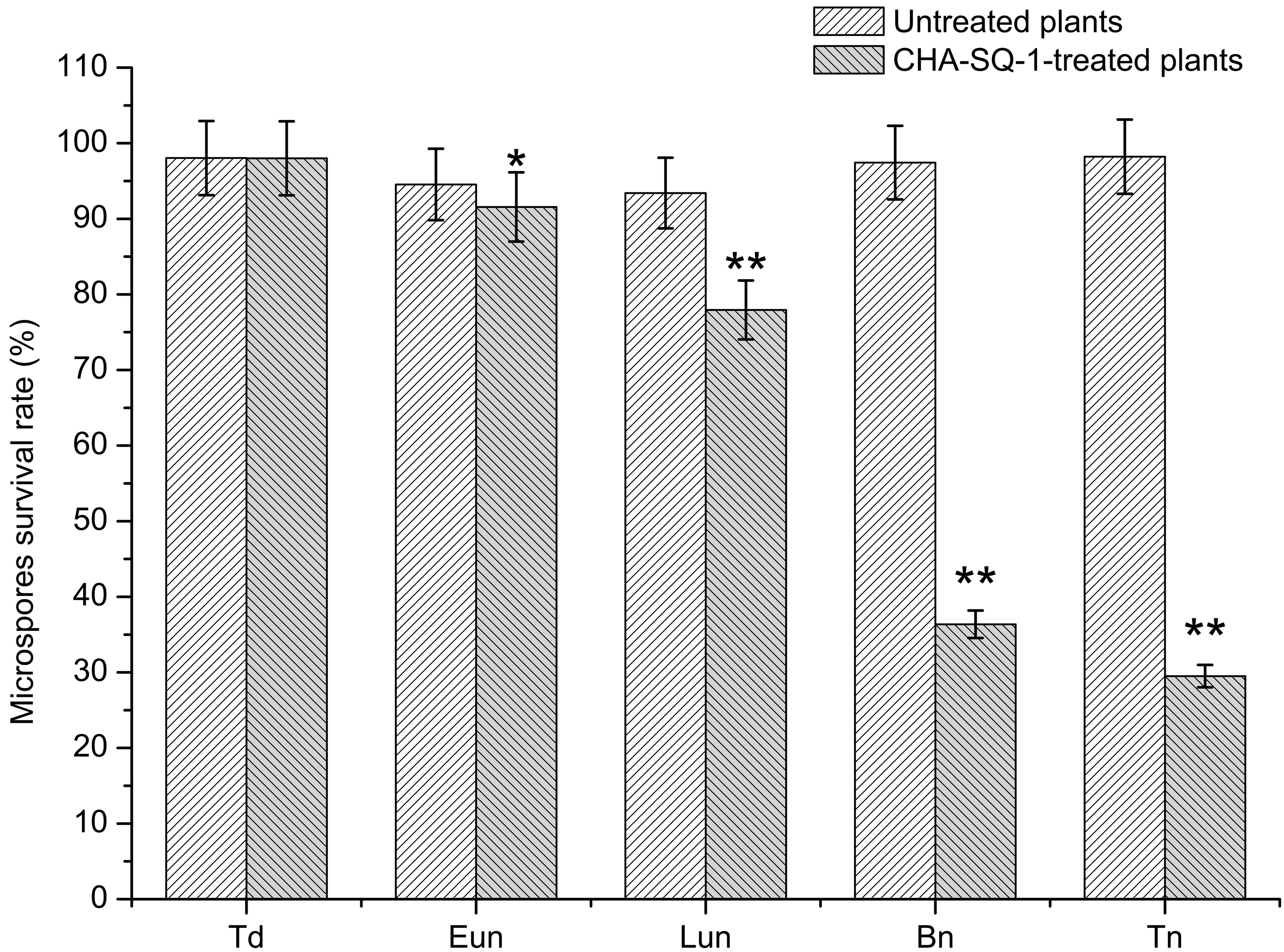

Supplement: S5 Fig — Microspores were stained with FDA (fluorescein diacetate) to determine cell viability and statistical analysis. Data which come from Fig. 6K to 6T and their repeated experiments images are means ± SD of three independent experiments. The significant of differences between untreated and CHA-SQ-1-treated plants were assessed by Student's t test (*P < 0.05, **P < 0.01). Td, Eun, Lun, Bn and Tn indicate the tetrad stage, the early-uninucleate, the later-uninucleate stage, the binucleate stage and the trinucleate stage, respectively. (TIF) [file pone.0119557.s005.tif]
